# Universal Genome Strand Parity Across Evolution

**DOI:** 10.3390/cells15201876

**Published:** 2026-10-15

**Authors:** Isabella Sbrana, Antonio Musio, Giulia Menconi

**Affiliations:** 1 Department of Biology, University of Pisa, 56100 Pisa, Italy; isabella.sbrana@unipi.it; 2 Institute of Biomedical Technologies (ITB), National Research Council (CNR), 56124 Pisa, Italy; antonio.musio@itb.cnr.it; 3 CISSC—Interdepartmental Center for Complex Systems Studies, University of Pisa, 56100 Pisa, Italy

**Keywords:** genome organization, strand parity of genes and transposons, fractal-like scaling behavior

## Abstract

**Highlights:**

**What are the main findings?**

- Eukaryotic genomes show universal DNA strand parity, with genes and all major TE classes occurring in nearly equal numbers and displaying highly similar length distributions. Gene and transposable element lengths follow power-law-like scaling, with nearly identical exponents on the two strands across all species.
- Strand parity extends to TE start positions, revealing conserved and highly symmetric structural patterns across evolution.

**What are the implications of the main findings?**

- Strand parity represents a previously unrecognized universal principle of genome organization. Power-law scaling of genomic element lengths supports a fractal-like genome architecture, linking local sequence symmetry to global structural constraints.
- Findings provide a structural explanation for Chargaff’s second rule, suggesting that oligonucleotide symmetry emerges from large-scale genomic parity.

**Abstract:**

The structural complexity of eukaryotic genomes remains a major open challenge in molecular biology. Genome architecture operates across multiple scales—from chromatin loops that mediate regulatory interactions, to isochores, chromatin domains and spatial territories. Genes within these regions are differentially distributed, replicated and expressed, underscoring the importance of long-range correlations for coordinated cellular functions. Here, we describe a previously unrecognized and evolutionarily conserved structural feature, the DNA strand parity, of eukaryotic genome. This principle governs the distribution of virtually all major genomic components. Across eleven evolutionary distant animal and plant species genes, exons, introns and all major transposable element (TE) classes occur in nearly equal number on the plus and minus DNA strands and display highly similar length distributions. The parity also extends to the start positions of TE fragments and to local sequence composition, as illustrated for LINE elements. This property provides a unifying explanation for longstanding observations of sequence-level symmetry, including the symmetry described by Chargaff’s second rule for single DNA strands. We further show that gene and TE lengths follow power-law-like distributions identical on either strand, with correlated scaling exponents across species, suggesting conserved fractal-like constraints during genome evolution. Together, these results establish strand parity as a universal principle of eukaryotic genome organization and support the view that it is an emergent property of the global fractal architecture of the genome.

## 1. Introduction

Genome organization in eukaryotes is governed by a hierarchical architecture that integrates chromatin interactions, sequence composition, and spatial nuclear compartmentalization. Investigations into chromatin–chromatin interactions within interphase nuclei have revealed the presence of A/B compartments—large, long-range interacting chromosomal regions shaped by the fractal globule model, which facilitates coordinated gene regulation. These compartments correlate with key genomic features, including gene density, chromatin activity, DNA accessibility, and histone modifications [1]. At higher resolution, topologically associating domains (TADs) have been defined as self-interacting chromosomal units, now recognized as fundamental building blocks of genome organization [2,3,4]. At even finer scales, dynamic chromatin loops mediate transient interactions between regulatory elements and gene promoters, with their length and stability modulated by cellular context. During mitosis, A/B compartments and TADs are no longer detectable; nonetheless, structural genome organization persists, as evidenced by chromosome banding patterns. R/G bands reflect distinct chromatin states and are associated with differential gene content (e.g., constitutive vs. tissue-specific), replication timing, and the spatial distribution of LINE and SINE transposons. These bands also align with GC-rich and GC-poor isochores—compositionally homogeneous DNA regions considered a fundamental layer of genomic structure [5]. However, with the advent of complete genome sequences in the early 21st century, it became evident that classical isochores, as originally defined, are not detectable in the human genome leading several authors to question its biological validity and empirical foundation [6,7,8]. Together, these observations underscore the high degree of order and non-randomness that governs genome architecture [9]. Despite substantial progress in elucidating the relationship between genome structure and function, many aspects remain unresolved. One complex issue is the Chargaff’s second rule is Chargaff’s second rule (Chargaff’s PR2), also known as the Symmetry Principle [10,11]. It posits that for any small oligonucleotide, the frequency approximates that of its reverse complement within the same DNA strand [12]. This principle holds across only the double-stranded eukaryotic, bacterial, viral and archaeal genomes, while single stranded DNAs not follow the rule. Evidence has been provided that it may reflect a deeper, evolutionarily conserved parity rule [13,14]. In this study, we describe a previously unrecognized property of genome architecture: strand parity in the number and size of genomic elements, observed across a broad phylogenetic spectrum. Specifically, we demonstrate that in all analyzed genomes (eleven species across distant evolutionary lineages), the frequency and length distributions of genes are strikingly similar between the plus and minus strands. This symmetry extends to other genomic features, including exons, introns, and all classes of transposable elements (TEs). A DNA sequence analysis may link this symmetry to the Chargaff’s PR2. Moreover, the distributions of genes and LINEs length on both strands conform to a shared power-law pattern across species. These findings suggest a solid relationship between genomic architecture and evolutionary dynamics, revealing a novel layer of genome organization rooted in strand-level symmetry and fractal-like scaling behavior.

## 2. Materials and Methods

We analyzed the genomes of eleven animal and plant species representing distantly related evolutionary taxa: *Homo sapiens* (Human), *Mus musculus* (Mouse), *Bos taurus* (Cow), *Monodelphis domestica* (Opossum), *Gallus gallus* (Chicken), *Xenopus tropicalis* (Xenopus), *Danio rerio* (Zebrafish), *Drosophila melanogaster* (Drosophila), *Caenorhabditis elegans* (*C. elegans*), *Arabidopsis thaliana* (Arabidopsis), and *Zea mays* (Maize). Scientific names were used at first mention and throughout all tables. In the main text, capitalized common names were used for readability, except for *Arabidopsis*, *C. elegans*, *Drosophila*, and *Xenopus*, in accordance with standard genetic nomenclature. Species names and assembly sources were listed in Supplementary Table S1.

Gene datasets were obtained from the NCBI Genome Annotation database [15], selecting the reference assembly for each species. An exception was *C. elegans*, whose genes—absent from NCBI—were retrieved from the UCSC Genome Browser [16]. Annotated genes included RefSeq-curated protein-coding (NM) and long non-coding RNA (NR) transcripts located on complete genomic molecules; predicted genes and genes on unplaced scaffolds were excluded. For *C. elegans*, only NM genes were included due to the lack of distinction among NR-type genes. Similarly, *M. domestica* and *X. tropicalis* contained only NM genes in the NCBI database. For each gene, only the representative transcript was analyzed, and chromosome coordinates were used to calculate gene sequence length. UCSC data were also used to define chromosome coordinates for G/R bands, exons, and introns. Coordinates for A/B compartments were derived from [17], and gene data for the hg19 assembly were retrieved from UCSC.

Datasets for TEs were obtained from the UCSC Genome Browser. For LINEs, SINEs, LTRs, and DNA transposons, all elements annotated in the RepeatMasker track were included without filtering; both complete and fragmented LINE elements have been included. DNA sequence length was calculated using start and end coordinates in the genomic sequence. TE start positions were defined using the internal coordinates within the repeat sequence.

Given the large number of observations per dataset (in some cases hundreds of thousands), analyses were performed using both individual sequence values and binned representations of the data. To characterize the global structure of sequence-length distributions, a rank–size representation was adopted. For each dataset, sequences were ordered by increasing length, and an integer rank was assigned to each observation. Rank–size plots were constructed by reporting sequence length (b) as a function of sequence rank. This representation allowed direct visual comparison of distributional patterns across strands and species without assuming a specific underlying probability distribution.

To further investigate the distributional properties of sequence lengths, empirical frequency distributions were computed by grouping sequence lengths into classes. For each dataset, sequences were assigned to bins defined over the observed length range; identical bins were used for gene lengths among vertebrates (15 kb), among other species (3 kb), and for LINE lengths among all species (200 b), enabling general comparison. The corresponding frequencies were used for statistical analyses. Given the substantial differences in absolute gene lengths across species, the frequency classes were ordered by increasing sequence length, and their ordinal position was used as a normalized index suitable for comparative purposes.

Log–log plots were constructed by reporting class frequencies as a function of class rank, defined as the ordinal index of each size class after ordering by increasing sequence length. This approach enabled comparison across datasets with heterogeneous length scales by focusing on relative ordering rather than absolute size. In the log–log representation, a subset of the distribution exhibited an approximately linear behavior over a large range of class ranks. This region was interpreted as a power-law-like scaling regime, indicating scale-invariant behavior over that interval. Linear regression analysis was performed within the identified scaling region to estimate the slope and the coefficient of determination ($R^{2}$), parameters used as descriptive measures of the extent and robustness of the scaling behavior.

The scaling exponents and line slopes obtained from single-strand values were used to compare the strands and thereby define their parity. The scaling exponents and slopes obtained from values aggregated across both strands were used to compare species and thus enable evolutionary interspecific comparisons.

Scaling extents were expressed in two complementary ways. The first, the rank-based extent, represented the number of decades covered on the rank axis; because this measure depends on the number of bins and may underestimate the true physical scale of the distribution, we also reported the size-based extent, in which the lower and upper bounds represented the size interval over which the power-law-like behavior held. This representation provided a descriptive characterization of the physical range over which scale-invariant behavior was maintained.

To investigate global patterns in the TE distribution of genomic start positions, rank–start-position plots were constructed, providing an intuitive, assumption-free visualization of their complex patterns across strands and species. Empirical frequency distributions were also computed, and their histograms were used for visual and statistical comparison.

Statistical analyses of gene and TE lengths, including chi-square tests and linear regression, were performed using STATGRAPHICS Centurion XVIII and Microsoft Excel. We defined statistical parity between datasets as cases where *p*-values exceeded $10^{-5}$, a threshold commonly accepted when comparing very large samples. This analytical framework enabled the identification of conserved distributional patterns across genomic datasets and provided a flexible approach for comparative analyses of large-scale genomic features.

## 3. Results

### 3.1. Gene Length Strand Parity Across Phylogenetically Distant Species

**Figure 1:**
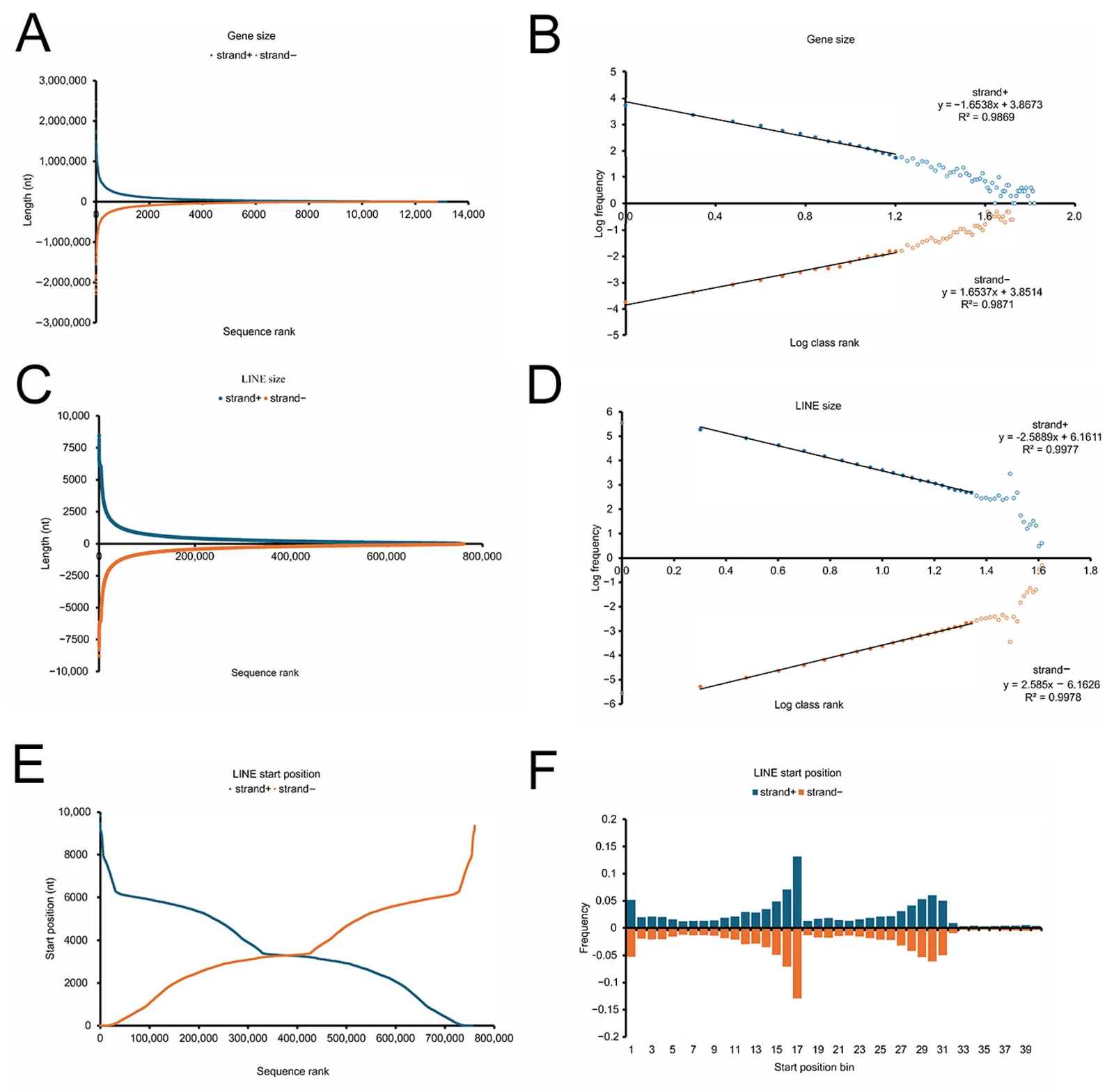
DNA strand parity for gene and LINE sequences in the human genome. (**A**,**C**) Rank– size plots of ordered gene and LINE sequence lengths in the Plus and Minus strands, with Minus-strand values plotted as negative Y-coordinates to visualize separately the two strands otherwise superposed. (**B**,**D**) Power-law-like scaling regimes of gene and LINE sequence lengths, inferred from the linearity in log–log space and quantified by linear regression line equations are reported to allow direct comparison between strands. (**E**) Rank–start-position plots of LINE start coordinate in both strands, with Plus-strand values plotted in descending order to visualize the two strands separately. (**F**) Frequency distributions of LINE start positions using 200 b-bins, with Minus-strand values plotted as negative Y-coordinates for optimal visualization. The 16–17 bins correspond to the size of 3200–3400 nt and the 29–32 bins correspond to the size of 5800–6400 nt.

Strand data were analyzed for eleven animal and plant species, spanning distant evolutionary lineages: Arabidopsis (*Arabidopsis thaliana*), *C. elegans* (*Caenorhabditis elegans*), Chicken (*Gallus gallus*), Cow (*Bos taurus*), Drosophila (*Drosophila melanogaster*), Human (*Homo sapiens*), Maize (*Zea mays*), Mouse (*Mus musculus*), Opossum (*Monodelphis domestica*), Xenopus (*Xenopus tropicalis*) and Zebrafish (*Danio rerio*) (Supplementary Table S1). Gene numbers on the plus and minus strands are approximately equal, and in some cases impressively almost identical as in Mouse (12,373 vs. 12,359), *Opossum* (10,867 vs. 10,804), *Drosophila* (7963 vs. 7968) (Supplementary Table S2). Gene length distributions substantially overlap between strands, with reverse-plotted curves revealing striking visual symmetry for all species (Figure 1A and Figure S1). In the human genome, the strand parity is evident not only at genome-wide but also within defined subregions including entire chromosomes, chromosomal subdomains such as G and R bands, and A/B epigenomic compartments. Furthermore, genes located on the opposite strands of chromosome 1, for example, exhibit comparable numbers of exons and introns with fully symmetrical length distributions (Supplementary Figure S2). Importantly, strand parity does not imply perfect one-to-one correspondence between strands; rather, it emerges as a robust statistical property, with only minor deviations (typically <1%). Such pervasive strand parity encompassing structural genomic elements across evolutionary distant taxa has not previously been documented.

### 3.2. Strand Parity Extends to Transposable Elements

**Figure 2:**
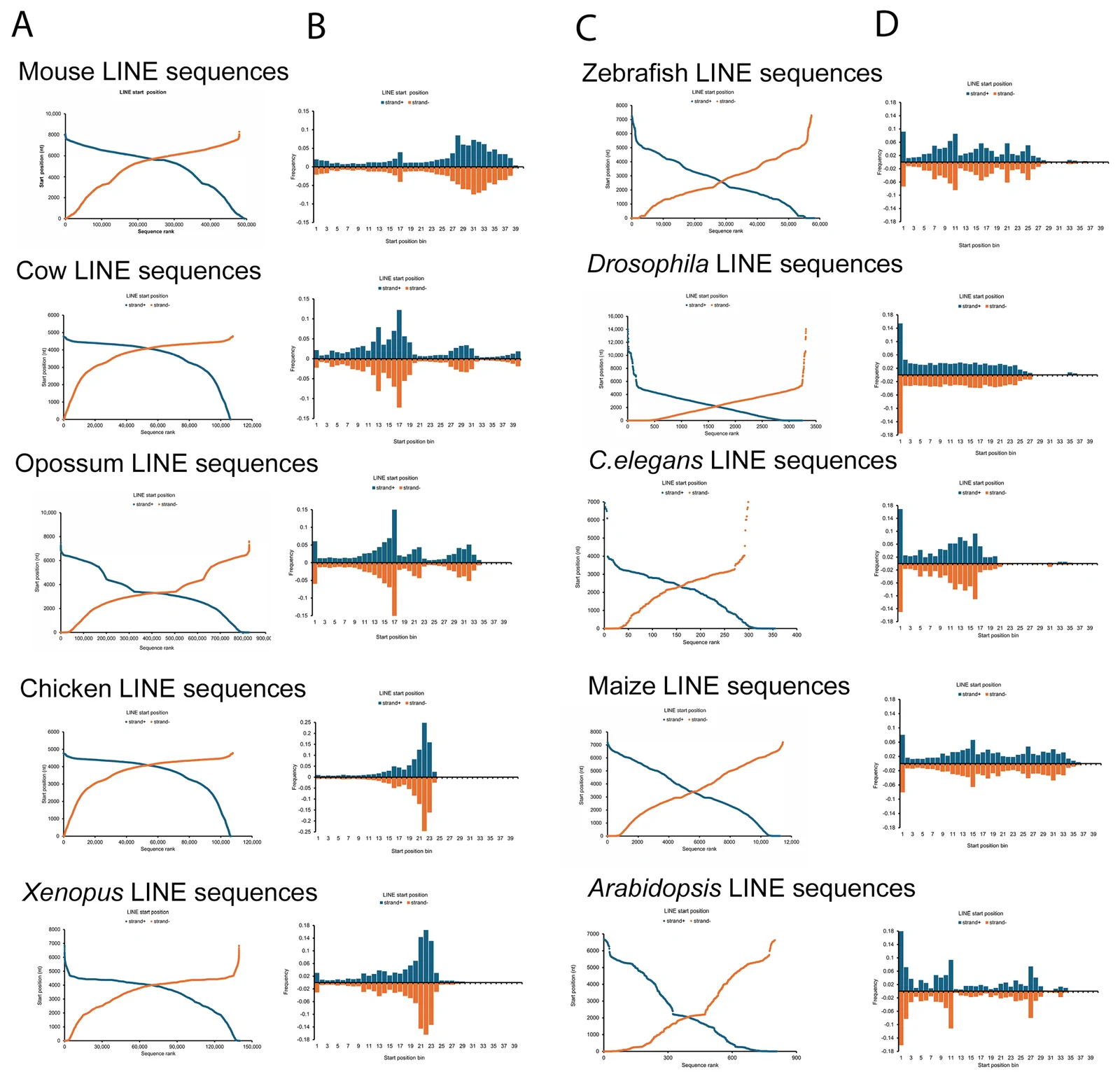
DNA strand parity for LINE sequence start positions across all species. (**A**,**C**) columns show rank–start-position plots of LINE start coordinate in the plus and minus strands, with plus-strand values plotted in descending order to visualize the two strands separately. (**B**,**D**) columns show frequency distributions of LINE start positions using 200 b-bins, with minus-strand values plotted as negative Y-coordinates for optimal visualization. The 16–17 bins correspond to the size of 3200–3400 nt and the 29–32 bins correspond to the size of 5800–6400 nt.

**Figure 3:**
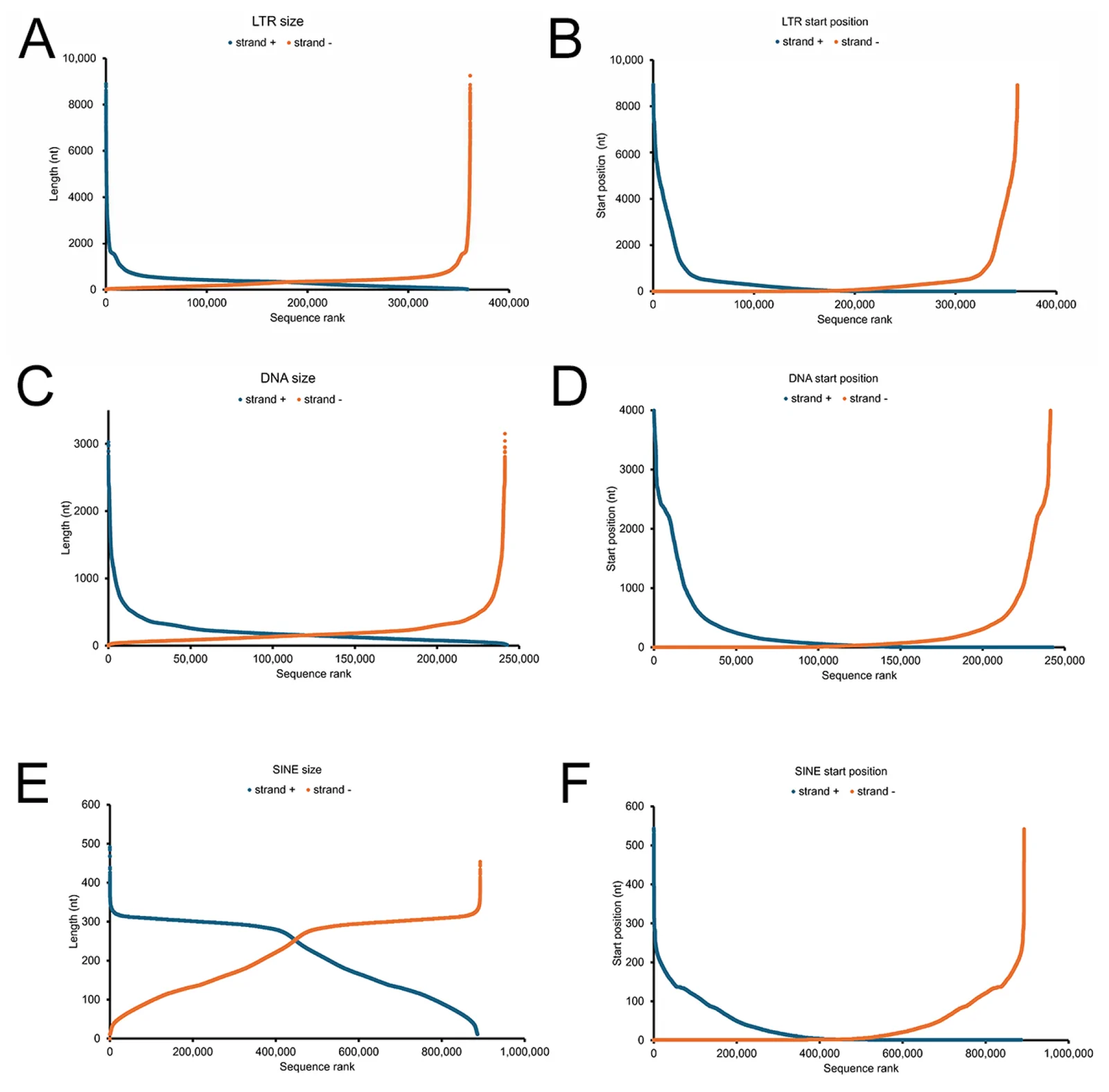
DNA strand parity for LTR, DNA, and SINE transposon sequences in the human genome. (**A**,**C**,**E**) Rank–size and (**B**,**D**,**F**) rank–start-position plots of ordered lengths of LTR, DNA, and SINE transposons in the plus and minus strands, with plus-strand values shown in descending order to visualize the two strands separately.

Transposable elements comprise roughly half of many eukaryotic genomes and are therefore major determinants of genomic structure [18]. LINEs, very ancient transposon that are ubiquitous across eukaryotes, exhibit, as observed for genes, full strand parity in number and length distribution encompassing full-length and fragmented elements across all species examined (Figure 1C and Figure S3). Nearly identical counts are observed in Opossum (1,268,157 vs. 1,268,230) and *Xenopus* (140,402 vs. 139,512), an impressive symmetry given the very large number of elements involved (Supplementary Table S2). Parity extends to the start positions, defined by UCSC annotations as the consensus sequence nucleotide at which each LINE element begins. To our knowledge, the distribution of these nucleotides in the genome is analyzed here for the first time. In humans, these start positions display a complex multimodal distribution that was nearly identical on the plus and minus strands, revealing a deep symmetry conserved in all species (Figure 1E,F and Figure 2). Their frequency-class analysis reveals prominent peaks in mammals (3200–3400 nt corresponding to bins 16–17 and 5800–6400 nt corresponding to bins 29–32). They show partially conserved positions in the other species, occurring on both strands with nearly perfect reciprocity. These peaks correspond to amplified fragments with shared start positions, likely reflecting the conservation of regions containing functional domains under positive selection. LINE elements have indeed been shown to harbor transcription factors and architectural protein binding sites, form secondary structures, and participate in long-range regulatory interactions [19,20]. Strand parity extends beyond LINEs to all other TE classes, including LTR, DNA, and SINE elements; again, nearly identical numbers and length distributions occur between strands across all species examined, with only minor taxon-specific deviations (Figure 3, Supplementary Table S3). Start position distributions also retain strand reciprocity in every TE class, indicating that the observed symmetry is not restricted to specific TE types but represented a global principle of genome organization.

In the human genome, the parity rule can be demonstrated even for all major LINE, LTR, DNA, and SINE transposon families, which show full strand symmetry in number, size, and start positions (Supplementary Figure S4). Furthermore, for the LINE-L1 family on human chromosome 22, the analysis of ATGC content reveals a clear identity between the two strands for fragments belonging to the same start position class. This pattern indicates, albeit indirectly, a substantial DNA sequence-level equivalence between corresponding fragments on opposite strands (Supplementary Figure S5).

### 3.3. Gene and Transposon Lengths Show Power-Law-like Distributions

Further key findings are that gene and LINE lengths follow almost identical power-law-like scaling on both strands. For genes by using identical homogeneous length classes (15 kb bins in vertebrates, 3 kb bins in other species) the log–log representation of their frequencies F as a function of class ranks r exhibited an approximately linear behavior over a wide range of class ranks, as determined by linear regression analysis (Supplementary Table S4A). In humans, the gene-length distributions on either strand follow the relation $F=k\cdot r^{\alpha }$ over 16 size classes, covering >95% of genes (Figure 1B). Scaling exponents α were nearly identical ($\alpha^{+}=-1.6538,\;\alpha^{-}=-1.6537$), as were the intercepts. Power-law-like scaling persists for genes within G/R bands, A/B compartments, and for exons and introns (Supplementary Figure S2). Comparable scaling exponents are consistently observed for the two strands across all other species, with high goodness of fit ($R^{2}>0.98$) (Supplementary Figure S1, Supplementary Table S4).

LINE elements (classes of 200 b bins) exhibit a similar pattern of power-law-like length scaling in log-log plots, showing matching exponents between strands and a high number of overlapping classes in all species (Figure 1D, Figure S3 and Supplementary Table S4B). In mammals, a second scaling region is observed on both strands, particularly pronounced in mouse. The primary scaling region substantially captures the size variation in the most LINE fragments, whereas the secondary region, ubiquitous in mammals and present also in *Xenopus*, corresponds to the distinct size variation in full-length LINEs. The strong correspondence of power-law exponents between the two strands provides formal evidence for the widespread strand parity in gene and LINE lengths.

### 3.4. Evolutionary Implications of Power-Law-like Scaling for Genome Structure

**Figure 4:**
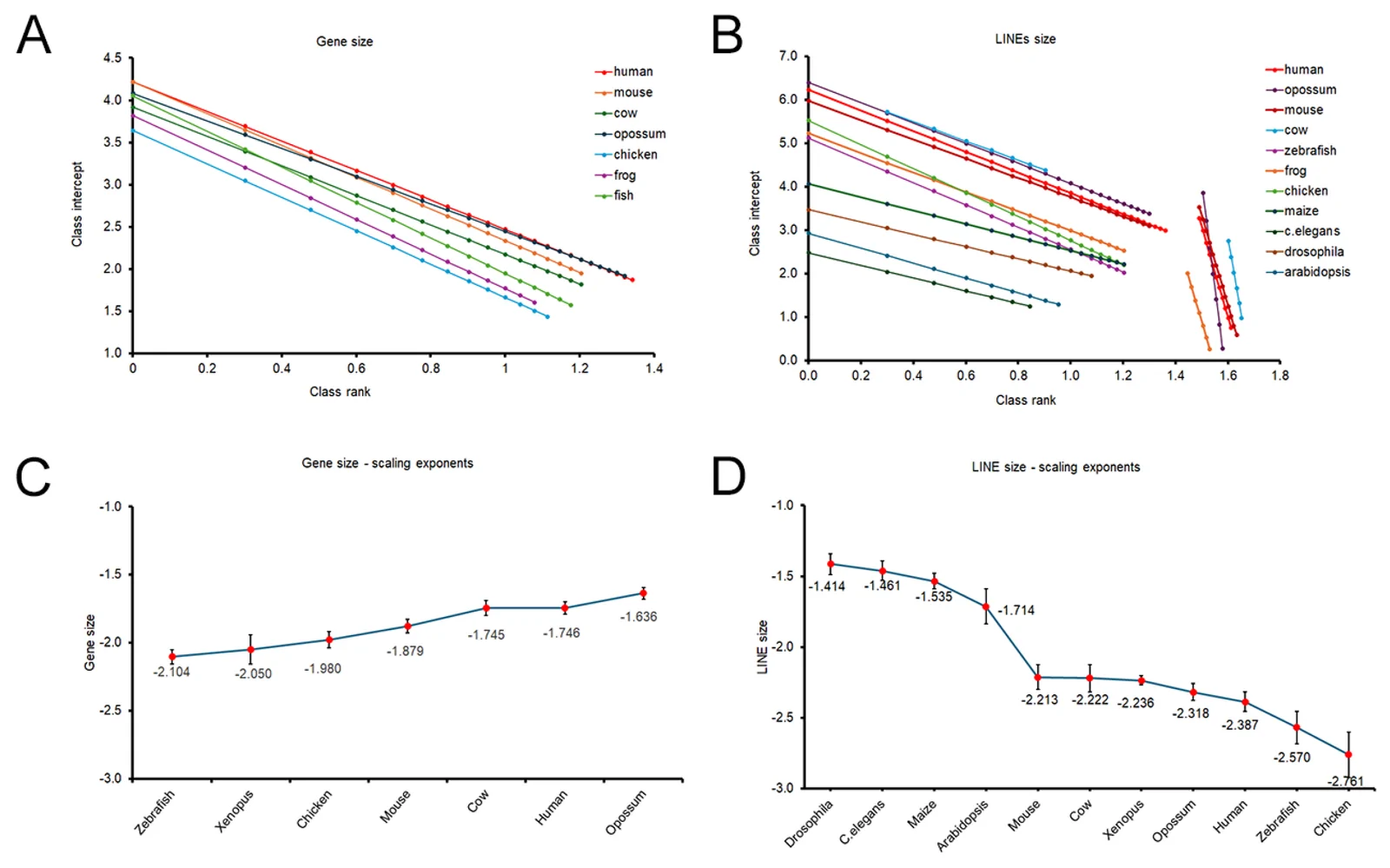
Gene and LINE size variation across genomes: comparison of power-law-like slopes and scaling exponents among species. Power-law-like slopes refer to values computed from linear regression on data aggregated across the two strands. (**A**) Gene size power-law-like slopes in vertebrate species, calculated using identical bin classes (15 kb-bins). (**B**) LINE size power-law-like slopes in all species (200 b-bins), shown for both the principal and secondary scaling regions. (**C**) Gene size scaling exponents and their standard errors, displaying an increasing trend across vertebrates, partially associated with genome complexity. (**D**) LINE size scaling exponents and their standard errors, showing a decreasing trend across species, with a clear distinction between vertebrates and the other species.

The power-law-like scaling analysis reveals additional general features. When comparing species, aggregating lengths from both strands to increase statistical power, the scaling exponents follow a pattern that we can interpret as an evolutionary gradient (Figure 4).

For genes, mammals show higher-magnitude scaling exponents from −1.6362 to −1.8791, consistent with broader size ranges; non-mammalian vertebrates exhibit reduced values from −1.9797 to −2.1041, whereas the other species display steeper slopes, from −2.0769 to −5.6131, and narrower size distributions (Supplementary Table S5A). Over evolutionary time, genomes underwent increases in gene number, size and complexity, driven by duplication and recombination of existing genes or gene fragments [21,22,23,24]. In this context, the shifts in scaling exponents observed across the analyzed vertebrates could be interpreted as indicators of gene size expansion along different evolutionary lineages. As the exponent increases (i.e., becomes less negative) from zebrafish to humans and opossums, the distribution decays more slowly, resulting in a more extended tail toward larger gene sizes. This pattern may reflect an evolutionary trend toward larger and more complex gene structures. From a biological perspective, the increase in the scaling exponent may provide a quantitative measure of the extent of gene size expansion across species. For LINEs, variation in the scaling exponent of the primary scaling region shows an opposite trend relative to genes, separating vertebrates, ranging from −2.2130 to −2.7611, from the other species, ranging from −1.4141 to −1.7138 (Supplementary Table S5B). From an evolutionary standpoint, these values likely represent the degree of LINE fragmentation, which was particularly pronounced in vertebrates. The limited variation observed in mammals reflects the partial structural modification of full-length elements, suggesting the involvement of distinct mechanisms.

Scaling exponents and extents are reported to reflect dense genome packing and self-similar, fractal-like spatial organization. Here, we quantified the scaling extent as a descriptive measure of the genomic intervals over which power-law behavior was observed for gene and LINE lengths. Because rank-based extents underestimated the underlying physical size range, we also provide size-based extents, defined by the lower and upper bounds of the distributions, for descriptive purposes. Gene lengths exhibit scaling over more than three orders of magnitude, whereas LINE elements span two orders of magnitude, consistent with their more restricted size range. These broad intervals indicate robust power-law behavior, as scaling over two to three decades, beyond statistical artefacts, is widely regarded as evidence of genuine self-similarity in natural systems [25,26]. Together, these results support a fractal-like organization of genomes.

## 4. Discussion

### 4.1. A Unified Framework for Genome Strand Parity

In this study, we identify strand parity as a universal principle of genomic architecture, encompassing all major genomic elements, both in terms of number and size, in phylogenetically distant eukaryotic from both animal and plant lineages. Its breadth implies deep evolutionary conservation over billions of years, sustained by genomic processes linked to strong selective pressure. A related and longstanding genomic feature is Chargaff’s PR2 which states that within any single strand of a double-stranded DNA molecule, the number of adenine and guanine is approximately matched by that of thymine and cytosine [10]. Later generalized as sequence or inverse symmetry, Chargaff’s PR2 has been documented in double-stranded prokaryotic and eukaryotic genomes, and extended to short oligonucleotides, which occur at frequencies closely matching those of their reverse complements. Our findings suggest that these symmetries, including Chargaff’s PR2, may reflect common genome-wide constraints rather than representing independent properties of DNA sequences. Because the reverse complement of any oligonucleotide corresponds to the same oligonucleotide on the opposite strand, Chargaff’s PR2 and the observed symmetry would be expected to arise if oligonucleotides were constrained to occur in equal numbers on both strands of the DNA duplex. Consistent with this idea, we find that LINE-L1 fragments, with defined start positions and full parity in both number and length, also display ATGC composition between strands, indicative of equivalent nucleotide sequence. This global parity in LINE-L1 fragment therefore provides a plausible structural basis for the parity described by the Chargaff’s PR2 and by oligonucleotide reverse complement equivalence. In this view, the strand-parity rule should share conceptual ground with Chargaff’s PR2 and related symmetry principles, for years extensively documented [25,27,28,29,30,31] as the DNA quadruplet symmetry [31].

### 4.2. Evolutionary Inferences

Power-law behavior has been documented in numerous biological context [32,33]. Genomic studies, although never focused on single DNA strands, have reported power-law distributions in exon spacers, transposons, conserved noncoding elements, $3^{\prime }$-untranslated regions, protein lengths, single-cell transcriptomes and related structures [34,35], proposing several models for their origin. In our analysis, the rank-frequency power-law relationship for gene and LINE length, while primarily descriptive, provides a robust basis for cross-strand and cross-species comparison. The resulting scaling exponent and extent values serve as proxies for the degree and strength of scale invariance, pointing to an underlying self-similar, fractal-like organization of the genome. Multiple lines of evidence support this interpretation. Both chromatin topology and sequence organization have been shown to exhibit hierarchical, scale-invariant patterns consistent with fractal-like genomic architecture, in agreement with the trends observed in our rank–frequency analysis. Empirical power-law relationships between genomic distance and contact probability in eukaryotic nuclei support the fractal globule model of chromatin folding, which describes a knot-free, dynamically accessible chromatin state that facilitates long-range regulatory interactions [1]. Power-law distributions of homologous repetitive elements, including LINEs, have likewise been proposed to stabilize this fractal conformation [36]. Fractal properties have also been identified directly in genomic DNA sequences, spanning repetitive elements, CpG islands, coding and non-coding regions, whole chromosomes, and complete genomes [27,32,37,38,39,40,41,42,43,44]. In this framework, the gene and TE length variation observed here may have been shaped by topological constraints imposed by fractal genome organization. Evolutionary increases in gene number, size, and structural complexity, arising from chromosomal duplication and rearrangement, would have been guided by such constraints to preserve large-scale chromosome organization, while additional evolutionary processes, such as TE transposition or fragmentation, would have followed the same pattern, possibly playing a role in the conformation [20,45,46,47]. One possible interpretation is that this symmetry reflects underlying dynamical constraints shaping genome architecture. If general fractal architecture provides a chromatin configuration that supports long-range regulatory interactions, specific topological features may diverge among species in relation to gene compositional complexity. In this sense the gradient in the scaling exponent may reflect the intrinsic capacity of genomes to generate structural complexity and so serve as a proxy for genome evolvability, by offering insights into the genomic elements and processes playing a role in evolutionary innovation [20,46]. Based on our comprehensive findings, we propose that strand parity is an emergent property of the universal fractal architecture of genomes, arising from topological constraints imposed by its functional organization. Such constraints would necessarily apply to all genomic elements irrespective of strand orientation, leading to parity as a natural consequence.

### 4.3. Functional Implications of Strand Parity

The evolutionary basis of strand parity remains to be fully elucidated, yet several mechanistic considerations suggest that this property may confer structural and functional advantages to eukaryotic genomes. First, a balanced distribution of genes and transposable elements between the two strands may stabilize chromosomal topology by minimizing asymmetric torsional stress during replication, transcription and chromatin folding. Such symmetry could support the maintenance of higher-order genome architecture, particularly within fractal-like chromatin domains where spatial packing constraints are strong. Second, strand parity may enhance replicative robustness. Comparable densities of functional and repetitive elements on both strands may reduce strand-specific replication delays, fork stalling and asymmetric mutational loads, thereby contributing to long-term genome stability. Third, a symmetric arrangement of transcription units may mitigate persistent conflicts between replication and transcription, a well-known source of genomic instability. Finally, strand parity may be maintained naturally from the cumulative effect of stochastic events such as inversions, segmental duplications and recombinations, which redistribute genomic elements across strands and preserve genomes in the equilibrium state. Importantly, this equilibrium does not reflect a thermodynamic minimization process, but rather a long-term evolutionary steady state emerging from the progressive neutralization of strand-specific compositional biases through recurrent genomic rearrangements. Experimental evidence indicates that disruptions of higher-order genome architecture, including its fractal-like organization, are a recurrent feature of human diseases. Mutations in *SMC1A*, *SMC3*, *NIPBL* and *CTCF* in cohesinopathies and cancer induce loss of local and global scaling properties of chromatin, and these changes precede transcriptional deregulation and genome instability, indicating a primary mechanistic role [48,49,50,51,52,53,54,55]. Multiple tumor types display loss of TAD integrity, aberrant compartment switching, and large-scale rewiring of chromatin interactions, all of which reflect a breakdown of the self-similar structural principles that normally govern genome folding. Such alterations are associated with transcriptional deregulation, replication stress, and increased genomic instability. In this context, the widespread strand parity described here may represent one of the structural constraints whose perturbation contributes to pathological chromatin states [56,57,58,59,60,61]. The conservation of strand parity across evolution suggests that it supports a stable and energetically favorable genomic configuration; its disruption, as observed in several malignancies, may therefore be part of the broader collapse of fractal genome organization that accompanies disease progression. Together, these considerations suggest that strand parity may represent an evolutionarily advantageous configuration that optimizes genome stability, regulatory efficiency and structural coherence across eukaryotic lineages.

## 5. Conclusions

Together, our findings reveal that DNA strand parity is a universal and deeply conserved feature of eukaryotic genomes, extending across genes, exons, introns and all major transposable element classes. The near-identical length distributions, start-position patterns and power-law-like scaling regimes observed on the two strands indicate that strand parity is not a local or taxon-specific phenomenon, but a global structural principle rooted in the fractal-like organization of the genome. This symmetry provides a unifying framework for understanding sequence-level regularities such as Chargaff’s second rule and suggests that strand parity emerges naturally from long-term evolutionary dynamics acting on large-scale genomic architecture. Overall, these results highlight strand parity as a fundamental constraint shaping genome structure across evolution.

## Data Availability

The data supporting the findings of this study are available in Article and its Supplementary Information, and from the corresponding authors on reasonable request.

## Supplementary Materials

The following supporting information can be downloaded at https://www.mdpi.com/article/10.3390/cells15201876/s1, Supplementary Table S1: Species names and assemblies of the eleven animal and plant species analyzed. Supplementary Table S2: Strand comparisons of gene and LINE numbers and length distributions across species. Supplementary Table S3: Strand comparisons of transposon numbers and length distributions across species. Supplementary Table S4: Strand comparison of scaling properties in gene and LINE distributions across species. Supplementary Table S5: Scaling properties of gene and LINE size distributions based on values aggregated across both strands in all species. Supplementary Figure S1: DNA strand parity for gene sequence length across all species. Supplementary Figure S2: DNA strand parity for gene, exon, and intron length within defined regions of the human genome. Supplementary Figure S3: DNA strand parity for LINE sequence size across all species. Supplementary Figure S4: DNA strand parity for each family of LTR, DNA, and SINE transposons in the human genome. Supplementary Figure S5: DNA strand parity for ATGC content in LINE-L1 elements of human chromosome 22.

## Abbreviations

The following abbreviations are used in this manuscript:

Chargaff’s PR2	Chargaff’s second rule
TADs	topologically associating domains
TE	transposable element
