# Supplementary material for: Universal Genome Strand Parity Across Evolution
Source: Cells. 2026 Oct 15;15(20):1876. doi: 10.3390/cells15201876 (PMC13702920; doi:10.3390/cells15201876)
Supplement: Supplementary file 1 [file cells-15-01876-s001.zip › cells-4590578-supplementary.pdf]

Supplementary Table S1. Species names and assemblies of the eleven animal and plant species analyzed.

Scientifc name	Name used in text	NCBI assembly	UCSC assembly
Arabidopsis thaliana	*Arabidopsis*	TAIR10.1	araTha1
Bos taurus	Cow	ARS- UCD2.0	bosTau9
Caenorhabditis elegans	*C.elegans*	--	ce11
Danio rerio	Zebrafish	GRCz11	danRer11
Drosophila melanogaster	*Drosophila*	ISO1MT	dm6
Gallus gallus	Chichen	bGalGal1	galGal6
Homo sapiens	Human	GRCh38.p14	hg38
Monodelphis domestica	Opossum	mMonDom1	monDom5
Mus musculus	Mouse	GRCm39	mm10
Xenopus tropicalis	*Xenopus*	UCB Xtro 10.0	xenTro10
Zea mays	Maize	Zm-B73	Zm-B73

Gene datasets were obtained from the NCBI Genome Annotation database, selecting the reference assembly for each species. Datasets for transposable elements (TEs) were obtained from the UCSC Genome Browser. Common names were also taken from the UCSC Genome Browser.

Supplementary Table S2. Strand comparisons of gene and LINE numbers and length distributions across species

		Plus Strand (n)	Minus Strand (n)	*(P)* - gene count	*(P)* - length frequency classes
(A)	**Genes**				
	Arabidopsis thaliana	15520	15403	0.5058	0.1360
	Bos taurus	7075	6817	0.0286	0.2188
	Caenorhabditis elegans	10049	9937	0.428	0.3550
	Danio rerio	7233	7441	0.086	0.1719
	Drosophila melanogaster	7963	7968	0.9684	0.3686
	Gallus gallus	3253	3100	0.0549	0.1992
	Homo sapiens	13171	12817	0.0281	0.1043
	Monodelphis domestica	10867	10804	0.669	0.5963
	Mus musculus	12373	12359	0.9291	0.6743
	Xenopus tropicalis	4164	4282	0.1992	0.1704
	Zea mays	9913	9823	0.5218	0.8300
(B)	**LINEs**				
	Arabidopsis thaliana	808	799	0.82236	0.7191
	Bos taurus	916247	923209	0.00000	0.5281
	Caenorhabditis elegans	355	299	0.02854	0.3825
	Danio rerio	58259	57293	0.00449	0.3020
	Drosophila melanogaster	3246	3313	0.40807	0.3740
	Gallus gallus	106444	107664	0.00837	0.8900
	Homo sapiens	755575	760651	0.00004	0.5206
	Monodelphis domestica	1268157	1268230	0.96344	0.4200
	Mus musculus	494775	479557	0.00000	$<10^{-5}$
	Xenopus tropicalis	140402	139512	0.09253	0.6176
	Zea mays	11269	11443	0.24827	0.0052

(a) Gene datasets include RefSeq-curated protein-coding (NM) and long non-coding RNA (NR) transcripts on genomic molecules. (b) LINE datasets include all full-length and fragmented annotated elements, without filtering. Gene datasets were sourced from the NCBI Genome Annotation database and include curated protein-coding (NM) and long non-coding RNA (NR) genes. LINE datasets were sourced from the UCSC Genome Browser. Statistics were performed by χ^2 test.

Supplementary Table S3. Strand comparisons of transposon numbers and length distributions across species

	Plus Strand (n)	Minus Strand (n)	*(P)* - transposon count	*(P)* - length frequency classes
**LTR**				
Arabidopsis thaliana	4,043	3,761	0.00141	0.0460
Bos taurus	262,501	265,642	0.00002	0.6466
Caenorhabditis elegans	356	317	0.13275	0.4623
Danio rerio	75,565	75,773	0.59288	0.4532
Drosophila melanogaster	7,321	7,889	0.00000	0.0769
Gallus gallus	19,755	19,366	0.04921	0.9370
Homo sapiens	358,821	361,356	0.00282	0.8408
Monodelphis domestica	302,184	302,302	0.87937	0.2230
Mus musculus	551,582	551,371	0.84077	0.0000
Xenopus tropicalis	30,203	29,700	0.03986	0.4496
Zea mays	429,690	432,903	0.00054	0.0000
**DNA**				
Arabidopsis thaliana	3,777	4,266	0.00000	0.0002
Bos taurus	165,299	164,994	0.59563	0.1141
Caenorhabditis elegans	16,827	16,752	0.68233	0.2864
Danio rerio	983,676	969,294	0.00000	0.0000
Drosophila melanogaster	2,184	1,827	0.00000	0.0001
Gallus gallus	18,414	17,419	0.00000	0.0022
Homo sapiens	242,673	241,321	0.05197	0.6063
Monodelphis domestica	152,504	152,241	0.63378	0.2236
Mus musculus	82,422	82,285	0.73569	0.2179
Xenopus tropicalis	680,145	667,520	0.00000	0.0000
Zea mays	93,475	94,864	0.00137	0.5712
**SINE**				
Arabidopsis thaliana	302	321	0.44653	0.5705
Bos taurus	1,048,162	1,048,310	0.91859	0.8706
Caenorhabditis elegans	243	288	0.05084	0.5804
Danio rerio	86,605	86,876	0.51528	0.2849
Drosophila melanogaster	-	-		
Gallus gallus	2,940	2,855	0.26417	0.3438
Homo sapiens	886,381	892,852	0.00000	0.0000
Monodelphis domestica	1,428,120	1,425,394	0.10658	0.5918
Mus musculus	747,410	745,936	0.22774	0.0880
Xenopus tropicalis	17,078	17,463	0.03831	0.7029
Zea mays	1,370	1,374	0.93913	0.2058

Transposon datasets include all full-length and fragmented annotated elements, with
transposon datasets were sourced from the UCSC Genome Browser database.
Statistics were performed by $\chi 2$ test.

Supplementary Table S4. Strand comparison of scaling properties in gene and LINE distributions across species

	Scaling exponent	Standard error	R^2	Rank classes	Scaling exponent	Standard error	R^2	Rank classes N.	Gene content covered by power-law (%)	
	Plus strand				Minus strand				Plus strand	Minus strand
(A) **Genes**										
*Arabidopsis thaliana**	-5.9351	0.1861	0.9961	6	-5.3882	0.1096	0.9983	6	22.28	23.36
*Caenorhabditis elegans**	-2.6779	0.1126	0.9843	11	-2.7084	0.1171	0.9835	11	99.52	99.70
*Drosophila melanogaster**	-2.1011	0.0462	0.9957	11	-2.0597	0.0487	0.9950	11	96.16	96.10
*Zea mays**	-2.8805	0.0624	0.9963	10	-2.9156	0.0675	0.9957	10	53.37	53.23
Bos taurus	-1.6969	0.0711	0.9810	13	-1.7622	0.0757	0.9801	13	94.33	94.90
Danio rerio	-2.1332	0.0847	0.9860	11	-2.0172	0.0746	0.9878	11	97.65	95.42
Gallus gallus	-1.9326	0.0667	0.9894	11	-1.9449	0.0873	0.9822	11	96.83	96.52
Homo sapiens	-1.6538	0.0508	0.9869	16	-1.6537	0.0505	0.9871	16	95.03	95.42
Monodelphis domestica	-1.5605	0.0496	0.9870	15	-1.5328	0.0352	0.9932	15	93.29	93.68
Mus musculus	-1.8601	0.0616	0.9870	14	-1.8635	0.0587	0.9882	14	95.91	96.29
Xenopus tropicalis	-1.9269	0.1587	0.9609	8	-1.9347	0.1393	0.9650	9	95.87	96.43
(B) **LINEs**										
Arabidopsis thaliana	-1.7727	0.1497	0.9524	9	-1.6675	0.1274	0.9607	9	87.78	86.54
Bos taurus	-2.2162	0.0958	0.9926	6	-2.2271	0.1001	0.9920	6	55.65	55.86
Caenorhabditis elegans	-1.5276	0.1023	0.9738	8	-1.4490	0.1099	0.9720	7	90.63	94.39
Danio rerio	-2.9074	0.1156	0.9844	12	-2.9678	0.1247	0.9826	12	43.67	44.1
Drosophila melanogaster	-1.3124	0.0564	0.9837	11	-1.4673	0.0942	0.9604	12	90.18	92
Gallus gallus	-3.2898	0.1061	0.9857	16	-3.2521	0.1038	0.9859	16	53.1	53.17
Homo sapiens	-2.5889	0.0286	0.9977	21	-2.5995	0.0276	0.9976	21	52.48	52.61
Monodelphis domestica	-2.5135	0.0265	0.9981	19	-2.5007	0.0248	0.9983	19	48.4	48.49
Mus musculus	-2.5248	0.0459	0.9944	19	-2.4541	0.0469	0.9935	20	60.5	59.31
Xenopus tropicalis	-2.2429	0.0338	0.9968	16	-2.2305	0.0343	0.9967	16	99.43	99.37
Zea mays	-1.4184	0.0580	0.9852	11	-1.4022	0.0625	0.9824	11	89.69	90.37

Scaling exponents were obtained from log-log regressions of rank-frequency data. (a) For genes the used class is 15kb-bins for vertebrate species and 3kb-bins for the other species (*), due to the very different sizes of genes. The two strands in all species show high linearity (R^2 > 0.98) except Xenopus. (b) For LINEs the class used for all species is 200b-bins. The two strands show high linearity (R^2 > 0.98) except in *C.elegans* and *Arabidopsis*.

Supplementary Table S5. Scaling properties of gene and LINE size distributions based on values aggregated across both strands in all species.

		Scaling exponent	Standard error	R^2	Rank classes	Scaling Range (Kb)	Rank-based Extent	Size-based Extent	Gene content covered by power-law (%)
(A)	**Genes**								
	Arabidopsis thaliana	-5.6131	0.0774	0.9992	6	3-21	0.544	0.845	22.85
	Caenorhabditis elegans	-2.9133	0.1154	0.9801	15	0.01-45	1.176	3.653	99.88
	Drosophila melanogaster	-2.0769	0.0367	0.9971	11	0.01-33	1.041	3.519	96.25
	Zea mays	-2.8948	0.0491	0.9977	10	3-27	0.740	0.954	53.32
	Bos taurus	-1.7449	0.0544	0.9866	16	0.1-240	1.204	3.380	96.16
	Danio rerio	-2.1041	0.0535	0.9917	15	0.1-225	1.176	3.352	98.86
	Gallus gallus	-1.9797	0.0600	0.9899	13	0.1-195	1.114	3.290	97.63
	Homo sapiens	-1.7461	0.0460	0.9863	22	0.1-330	1.342	3.519	97.14
	Monodelphis domestica	-1.6362	0.0431	0.9870	21	0.1-315	1.322	3.498	96.53
	Mus musculus	-1.8791	0.0510	0.9898	16	0.1-240	1.204	3.380	96.82
	Xenopus tropicalis	-2.0499	0.1091	0.9724	12	0.1-180	1.079	3.255	98.30
(B)	**LINEs**								
	Arabidopsis thaliana	-1.7138	0.1246	0.9643	9	0.01-1.8	0.954	2.255	86.86
	Bos taurus	-2.2216	0.0979	0.9923	6	0.2-1.6	0.544	0.903	52.29
	Caenorhabditis elegans	-1.4610	0.0669	0.9896	7	0.01-1.4	0.845	2.146	90.36
	Danio rerio	-2.5699	0.1129	0.9737	16	0.01-3.2	1.204	2.505	99.20
	Drosophila melanogaster	-1.4141	0.0731	0.9740	12	0.01-2.4	1.079	2.380	91.60
	Gallus gallus	-2.7611	0.1605	0.9548	16	0.01-3.2	1.204	2.505	98.87
	Homo sapiens	-2.3875	0.0678	0.9833	23	0.01-4.6	1.362	2.663	99.25
	Monodelphis domestica	-2.3184	0.0605	0.9879	20	0.01-4.0	1.301	2.602	98.97
	Mus musculus	-2.2130	0.0858	0.9737	20	0.01-4.0	1.301	2.602	91.74
	Xenopus tropicalis	-2.2360	0.0317	0.9972	16	0.01-3.2	1.204	2.505	99.41
	Zea mays	-1.5355	0.0549	0.9824	16	0.01-3.2	1.204	2.505	94.00

(a) For genes the vertebrate species (class 15kb-bins) and the other ones (class 3kb-bins) are reported separately. Sizes refer to values aggregated across both strands. For comparative purposes, in addition to the Extent computed on ranks, the true physical range of sizes and the corresponding size-based Extent are reported. (b) For LINEs the rank used for all species is 200b-bins.

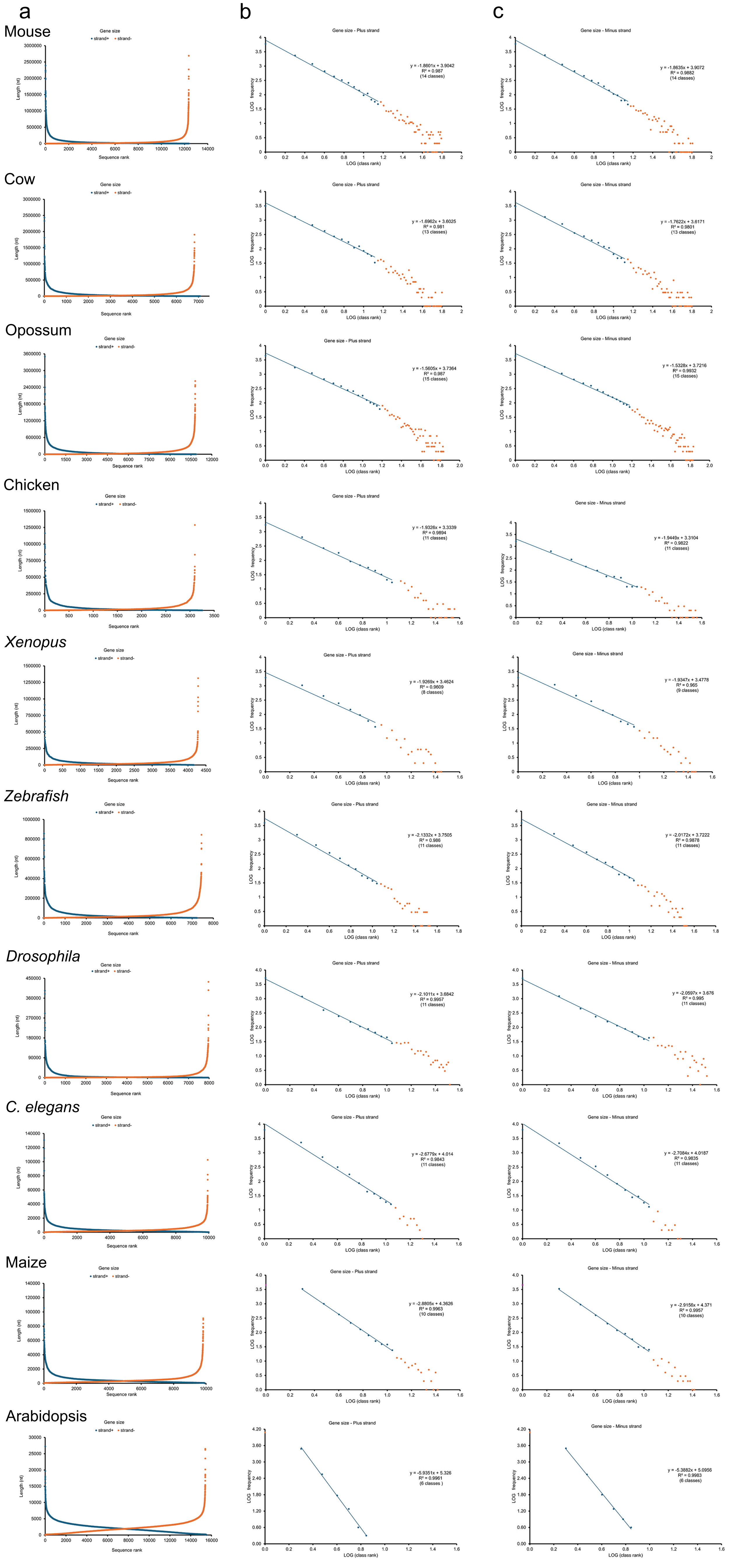

Supplementary Figure S1

Supplementary Figure S2

a

Gene sequence lengths on single chromosomes

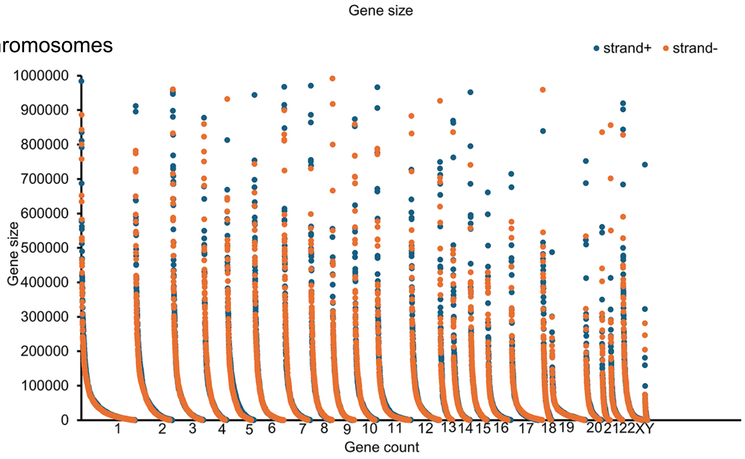

b

Gene sequences in G-type chromosome bands

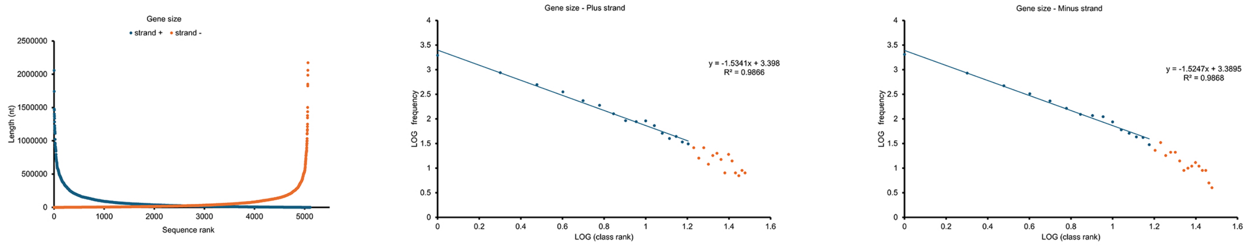

c

Gene sequences in R-type chromosome bands

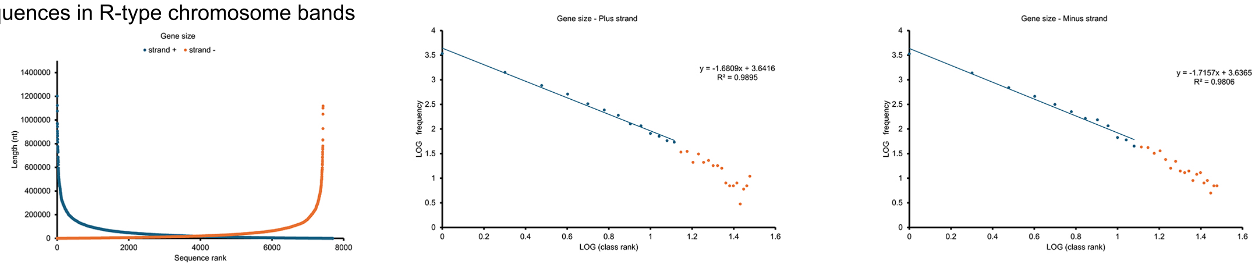

d

Gene sequences in A-type compartments

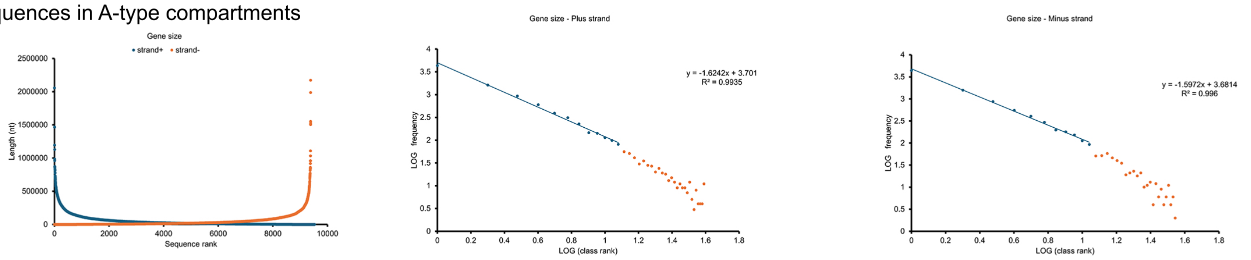

e

Gene sequences in B-type compartments

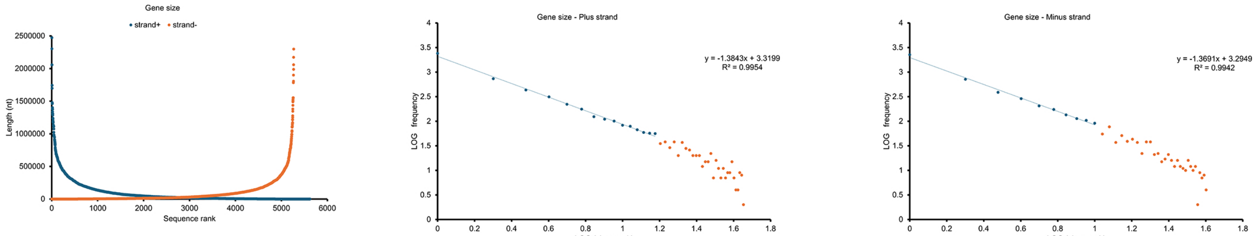

f

Exon sequences on chromosome 1

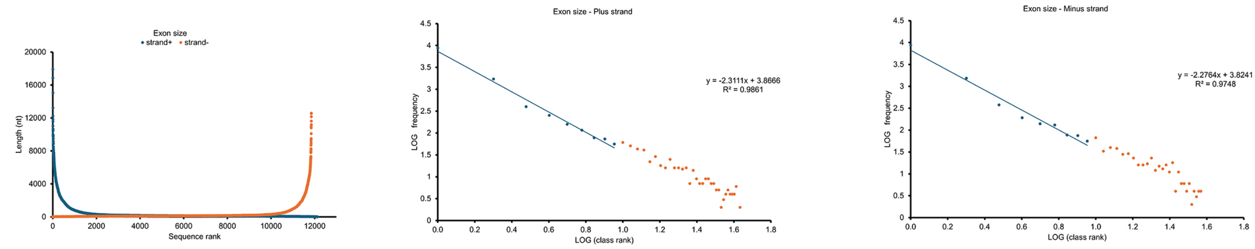

g

Intron sequences on chromosome 1

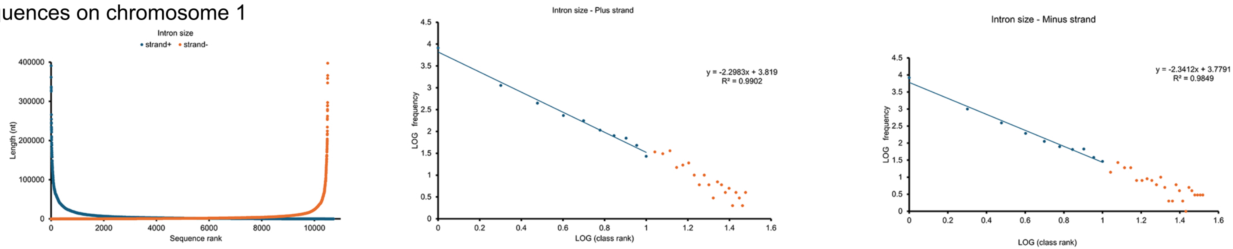

Supplementary Figure S3

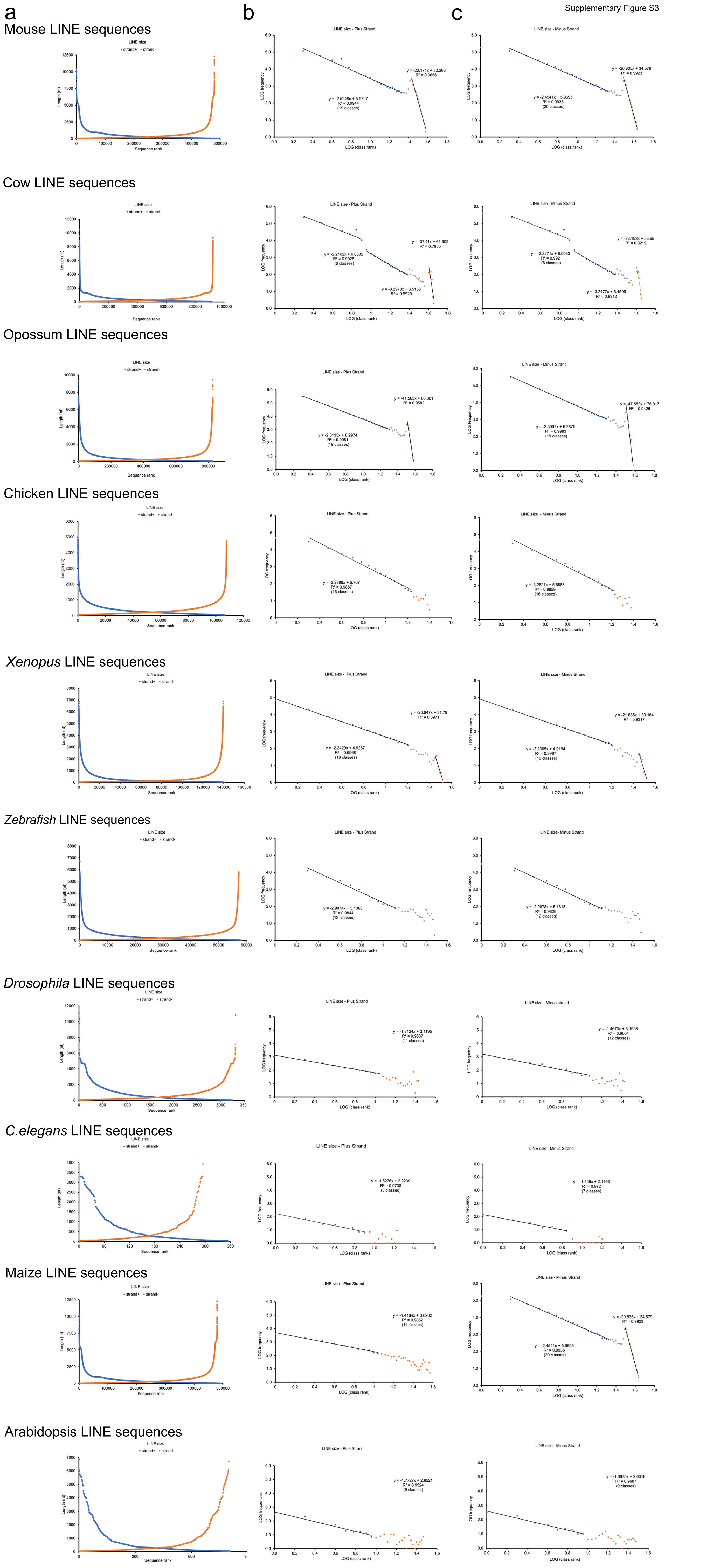

Supplementary Figure S4

a

LINE transposon families

Family	N. strand+	N. strand-
L1	478,110	483,975
L2	231,229	230,548
CR1	33,336	33,293
RTE-X	7,643	7,553
RTE-bovB	4,461	4,476
Penelope	532	543
Dong-R4	264	263

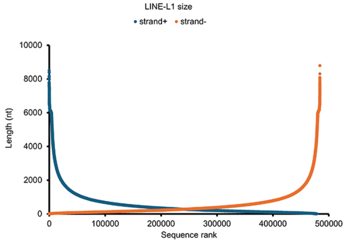

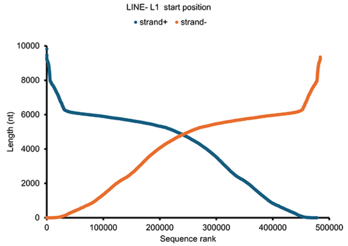

b

SINE transposon families

Family	N. strand+	N. strand-
Alu	587,625	593,447
MIR	293,281	294,033
tRNA & similar	4,136	4,194
5S -Deu-L2	1,339	1,178

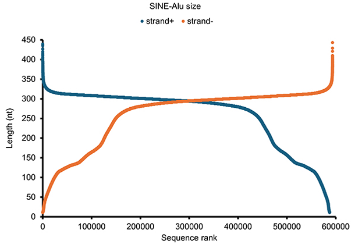

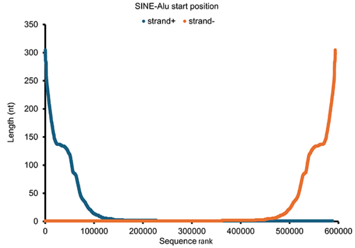

c

LTR transposon families

Family	N. strand+	N. strand-
ERVL-ERVLMaLR	253,885	254,734
ERV1	86,260	87,599
Gypsy	12,000	11,966
ERVK	5,143	5,457
LTR	1,533	1,600

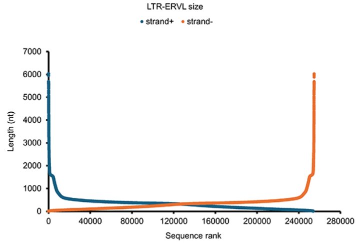

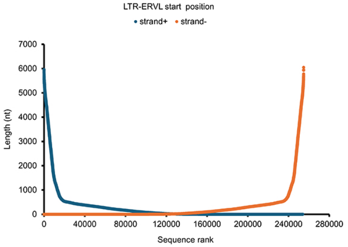

d

DNA transposon families

Family	N. strand+	N. strand-
hAT	168,985	168,170
TcMar	70,283	69,916
DNA	1,153	1,126
PiggyBac	1,223	1,117
MULE	993	939
Merlin	22	38
Harbinger	14	15

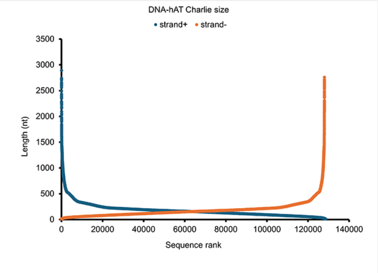

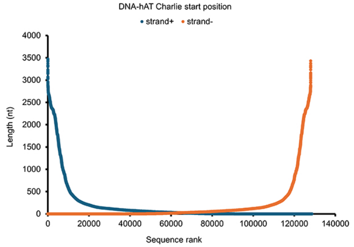

Supplementary Figure S5

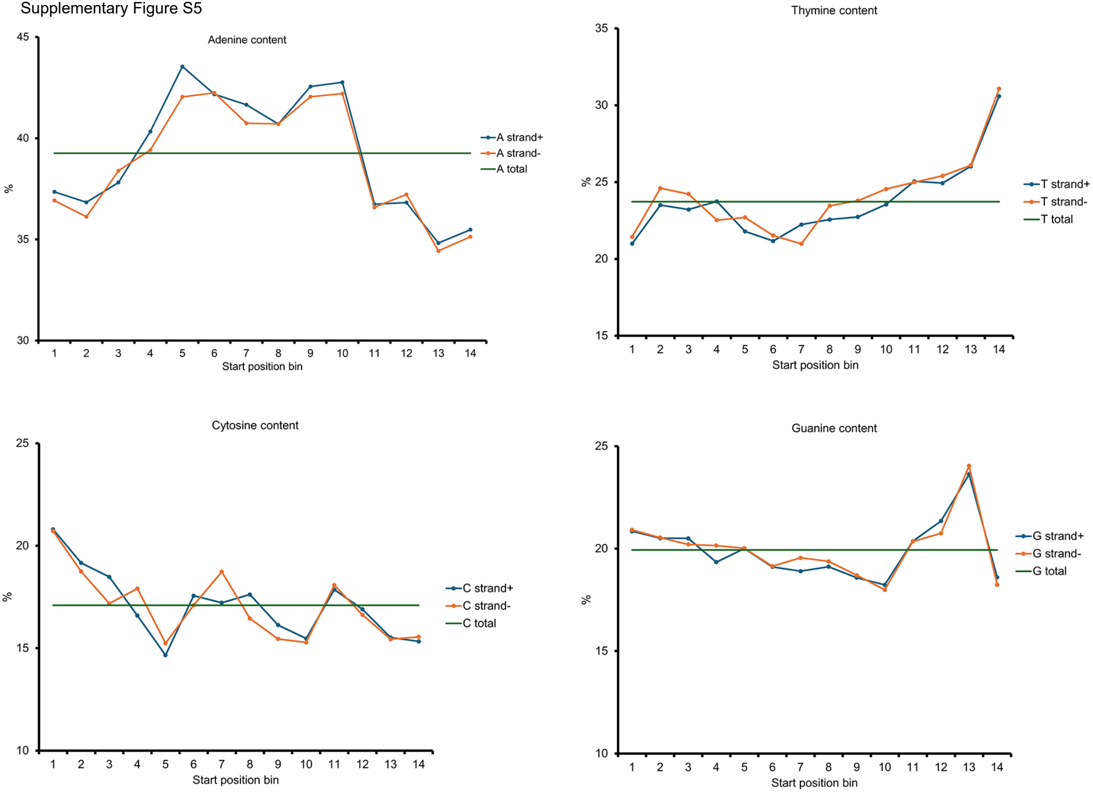

SUPPLEMENTARY FIGURE LEGENDS

Supplementary Figure S1 DNA strand parity for gene sequence length across all species. a, Rank–size plot of ordered gene sequence lengths in the Plus and Minus strands, with Plus-strand values plotted in descending order to visualize the two strands separately. **b–c,** Power-law-like scaling regimes of gene sequence lengths in each strand, inferred from linearity in log-log space and quantified by linear regression. Line equations are reported to allow direct comparison between strands.

Supplementary Figure S2 DNA strand parity for gene, exon, and intron length within defined regions of the human genome. a–e, Gene-size scatter plots and power-law distributions in the Plus and Minus strands within: individual chromosomes, G- and R-bands, A/B compartments of the human genome (coordinates for compartments and related genes from the UCSC hg19 assembly. **f–g,** Exon and intron size scatter plots and power-law distributions within human chromosome 1. Line equations are reported to allow direct comparison between strands.

Supplementary Figure S3 DNA strand parity for LINE sequence size across all species. a, Rank–size plots of ordered LINE sequence lengths in the Plus and Minus strands, with Plus-strand values plotted in descending order to visualize the two strands separately. **b–c,** Power-law-like scaling regimes of LINE sequence lengths in each strand, inferred from linearity in log–log space and quantified by linear regression. Line equations are reported to allow direct comparison between strands. In Mouse, an exception to strand parity occurs in the 900–1000 bp class (red dot) of the Plus strand, where observations are three times more frequent than in the Minus strand, affecting statistical comparisons. This excess primarily involves the L1Md subfamily, which accounts for 83.12% of all elements in this class. In Cow, a deviation from the expected power-law pattern occurs in the 1200–1400 bp class (red dot), where frequencies on both strands are more than twice the expected values; this deviation is entirely attributable to the RTE-Bov subfamily.

Supplementary Figure S4 DNA strand parity for each family of LTR, DNA, and SINE transposons in the human genome. a, Tables reporting the presence of all human transposon families in the

Plus and Minus strands. **b–c,** Rank–size and rank–start-position plots of ordered lengths of LINE, LTR, DNA, and SINE family elements in the Plus and Minus strands, with Plus-strand values plotted in descending order to visualize the two strands separately.

Supplementary Figure S5 DNA strand parity for ATGC content in LINE-L1 elements of human chromosome 22. Comparison of adenine, thymine, cytosine, and guanine content in the Plus and Minus strands of LINE-L1 elements on human chromosome 22, grouped into frequency classes based on start positions. These classes correspond to those showing differential frequencies in the human genome (Figure 1).
